# Real-world use of intravenous ferric derisomaltose in Brazilian patients with iron deficiency anemia

**DOI:** 10.1016/j.htct.2026.106501

**Published:** 2026-07-20

**Authors:** Lauro Augusto Caetano Leite, Gisele dos Santos Barros, Thomas Azevedo do Carmo, Rodolfo Delfini Cançado

**Affiliations:** a Faculdade de Ciências Médicas da Santa Casa de São Paulo, São Paulo, Brazil; b Hospital Samaritano-Higienópolis, São Paulo, Brazil; c Hospital São Paulo – UNIFESP, São Paulo, Brazil

**Keywords:** Iron deficiency anemia, Intravenous iron, Ferric derisomaltose, Hemoglobin, Hypophosphatemia, Oral iron intolerance

## Abstract

**Background:**

Iron deficiency anemia remains a major global health concern, especially among women, children, and people with chronic illnesses. Oral iron therapy is the standard treatment, but it is often limited by gastrointestinal side effects or poor absorption. Intravenous ferric derisomaltose is a newer formulation that allows high-dose, rapid iron replenishment with a favorable safety profile. This study aimed to assess the effectiveness and safety of intravenous ferric derisomaltose in patients with iron deficiency anemia who could not tolerate or did not respond to oral iron.

**Methods:**

This was a prospective, open-label, single-center trial conducted in São Paulo, Brazil. Adult patients (≥18 years) with iron deficiency anemia received 1 or 2 infusions of intravenous ferric derisomaltose (up to 1000 mg per infusion). Hematologic and iron parameters were measured at baseline, Week 4, and Week 8. Safety outcomes included adverse events and serum phosphate levels.

**Results:**

Thirty patients were treated with 25 requiring a second infusion. Significant increases in hemoglobin, serum ferritin, transferrin saturation, and reticulocyte hemoglobin content were seen at both time points. By Week 8, 81% achieved a hemoglobin increase ≥2 *g*/dL, and 54% achieved an increase of ≥3 *g*/dL. Four (8%) mild adverse events occurred and three patients (10%) experienced transient, asymptomatic hypophosphatemia, which resolved without intervention.

**Conclusion:**

This study contributes to the growing evidence supporting the use of intravenous ferric derisomaltose as a safe and effective alternative for distinct populations with iron deficiency anemia. In this real-world clinical setting, the treatment is effective for treating patients with iron deficiency, providing rapid hematologic recovery with a favorable safety profile.

## Introduction

As the most prevalent hematologic disorder globally, anemia affects approximately 1.8 billion individuals; among these cases, iron deficiency anemia (IDA) accounts for nearly 50% of the total burden [1]. IDA can be caused by inadequate dietary intake, poor absorption, ongoing blood loss, or high iron requirements. It has a tremendous public health significance affecting children, adolescents and women of reproductive age worldwide [1, 2, 3, 4].

Historically, oral iron supplementation has been recommended as the first-line therapy for IDA, especially in outpatient and resource-limited settings [1, 2, 3, 4]. Gastrointestinal side effects, including nausea, constipation, abdominal discomfort, and diarrhea, occur frequently and contribute to poor adherence in up to 40% of treated patients, thereby limiting its effectiveness in real-world practice [5]. Additionally, inflammation, gastrointestinal disorders, previous bariatric surgery, or ongoing blood loss can hinder oral iron absorption and reduce treatment efficacy [2,3,5]. In this context, intravenous (IV) iron replenishes iron stores rapidly, thereby quickly raising hemoglobin (Hb) levels, and thus has gained recognition as an effective and well-established treatment for patients who are intolerant of, unresponsive to, or unsuitable candidates for oral iron supplementation [1, 2, 3, 4]. IV Ferric Derisomaltose (IFD) is a newer drug capable of delivering high doses of iron in a single or a few infusions [6]. This approach benefits patient convenience and helps ensure compliance, ultimately improving treatment outcomes [6,7]. The immediate and long-term effects of IFD on Hb and iron stores have been documented in both clinical trials and real-world studies, showing notable safety advantages and few hypersensitivity reactions [6, 7, 8]. Furthermore, randomized comparative trials suggest that the risk of clinically significant hypophosphatemia with IFD is lower than with other IV iron formulations [8]. Pharmacoeconomic analyses demonstrate a balance among efficacy, safety, and cost, especially in healthcare systems aiming to maximize efficiency while maintaining high-quality care [8, 9, 10]. These findings supported the inclusion of IV iron therapies, including IFD, in international guidelines for iron deficiency and IDA across various clinical conditions [9, 10, 11].

Given the global prevalence of IDA and the limitations of oral therapy, and as the evidence supporting IV agents continues to grow, this study aims to assess the effectiveness and safety of IFD in adults with IDA.

## Methods

### Study design and ethical approval

This trial was a prospective, open-label, single-center clinical study of IFD treatment in patients with IDA who were intolerant of, or unresponsive to oral iron therapy. Unsatisfactory response to oral iron was defined as a failure to achieve an increase of 1 g/dL in Hb concentration from baseline after at least four weeks of treatment (100–200 mg/day of oral iron) in the previous 12 months. Oral iron intolerance was defined as the inability to complete treatment due to gastrointestinal symptoms, including nausea, abdominal pain, constipation, or diarrhea [9]. The etiology of IDA was determined based on the patient’s clinical history (e.g., heavy menstrual bleeding) and investigations into underlying causes, including upper and lower gastrointestinal endoscopy and H. pylori assessment [9]. The study was conducted at Hospital Samaritano de São Paulo, Brazil, from May 2023 to December 2023, in accordance with the Declaration of Helsinki and good clinical practice guidelines. Ethical approval was obtained from relevant ethics committees before starting study, and all participants provided written informed consent.

### Inclusion and exclusion criteria

Participants were adults (≥18 years) diagnosed with IDA, defined as Hb levels below 12 g/dL for females and below 13 g/dL for males, and serum ferritin <30 ng/mL or <100 ng/mL if transferrin saturation (TSAT) <20%. Patients with active infections, chronic liver disorders, known hypersensitivity to IV iron, a history of blood transfusion, or severe conditions requiring urgent hospital treatment were excluded. Diagnostic criteria were strictly adhered to in accordance with internationally recognized guidelines [9,12].

### Intervention and dosage

Treatment involved administering a single IV injection of IFD (Monofer®; Pharmacosmos) based on body weight and baseline Hb levels, following the dosing matrix used in recent clinical trials [12, 13, 14, 15]. Specifically aligning with Food and Drug Administration (FDA) recommendations, the maximum infusion dose was limited to 1000 mg over 15–30 min in outpatient settings [7,12,13]. When necessary, a second dose of 500 or 1000 mg was administered seven days after the first. Baseline blood tests, including Hb, mean corpuscular volume (MCV), serum ferritin (SF), TSAT, reticulocyte Hb content (Ret-He), and serum phosphate level, were performed before the first infusion, with follow-up tests at Weeks 4 and 8. Participants were observed during infusion and for 30 min afterward for signs of hypersensitivity reactions., [13,14].

## Statistical analysis

Continuous variables are presented as medians with interquartile ranges (IQRs). Clinical groups were compared using the Kruskal-Wallis test. Statistical significance was defined as p-values <0.05. All analyses and figures were generated using GraphPad Prism.

### Primary and secondary outcomes

The primary outcome was the change in Hb level from baseline to Weeks 4 and 8. Secondary outcomes included the percentage of patients achieving an improvement in Hb ≥2 g/dL and ≥3 g/dL; as well as the incidence and types of adverse events (AEs), including hypophosphatemia (serum phosphate <2mg/dL).

## Results

### Patient characteristics

A total of 30 participants were enrolled in the study; 28 (93.3%) were female. The mean age was 48.2 years (range: 23–83), with a median of 46 years. Regarding the reason for treatment, 23 (76.6%) had an unsatisfactory response to oral iron, and seven (23.3%) had intolerance to oral iron. Among the 28 women, 15 (53.3%) experienced abnormal uterine bleeding associated with uterine myoma. Six (20%) of the 30 patients had gastrointestinal bleeding, and eight (26.6%) had a history of bariatric surgery. Among the two male participants in this cohort, the main reason for iron deficiency was gastrointestinal bleeding.

The 30 participants received an initial infusion of 1000 mg of IFD. Of the twenty-five who required a second infusion, fifteen received an additional 1000 mg, and ten received 500 mg, based on iron-deficiency estimates.

### Efficacy

**Fig. 1:**
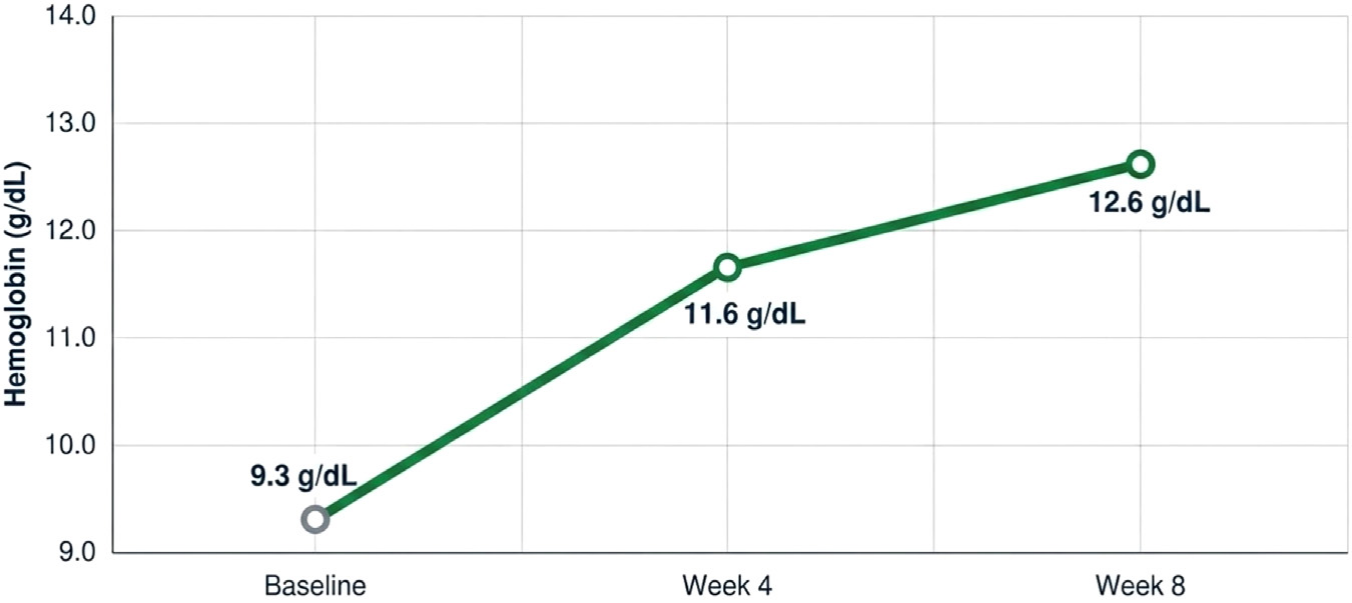
Hemoglobin trajectory illustrating rapid and complete normalization by Week 8.

**Fig. 2:**
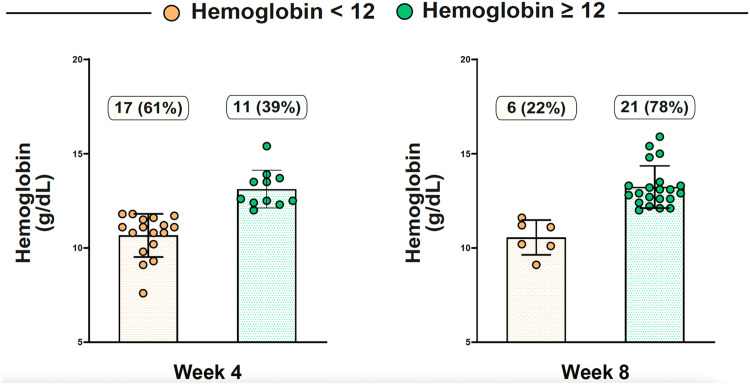
Distribution of patients achieving Hb ≥12 *g*/dL vs. <12 *g*/dL from baseline to Week 8.

**Fig. 3:**
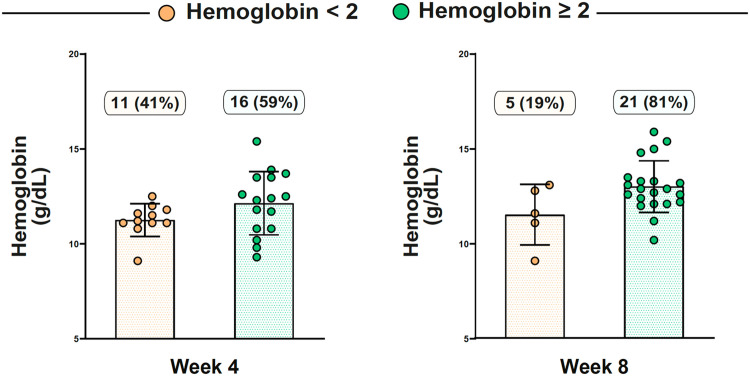
Distribution of patients achieving increases of ≥2 *g*/dL vs. <2 *g*/dL of hemoglobin from baseline to Weeks 4 and 8.

**Fig. 4:**
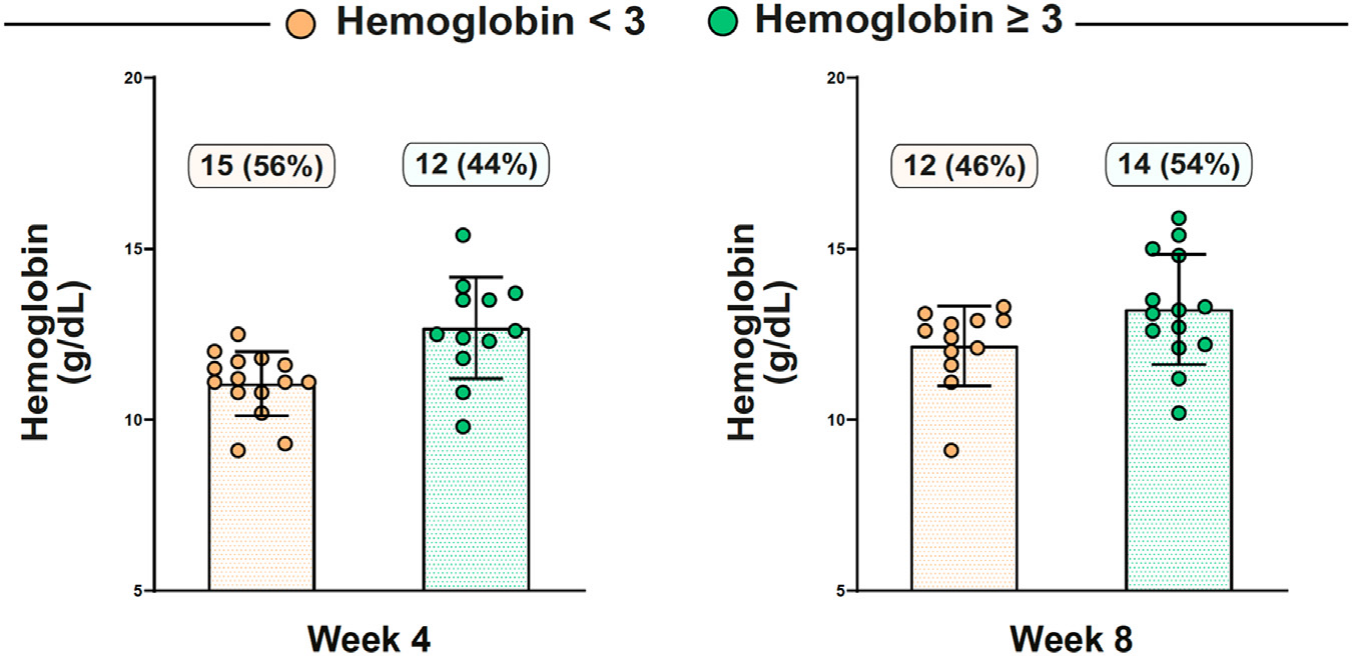
Distribution of patients achieving an increase ≥3 *g*/dL vs. <3 *g*/dL of hemoglobin from baseline to Weeks 4 and 8.

**Fig. 5:**
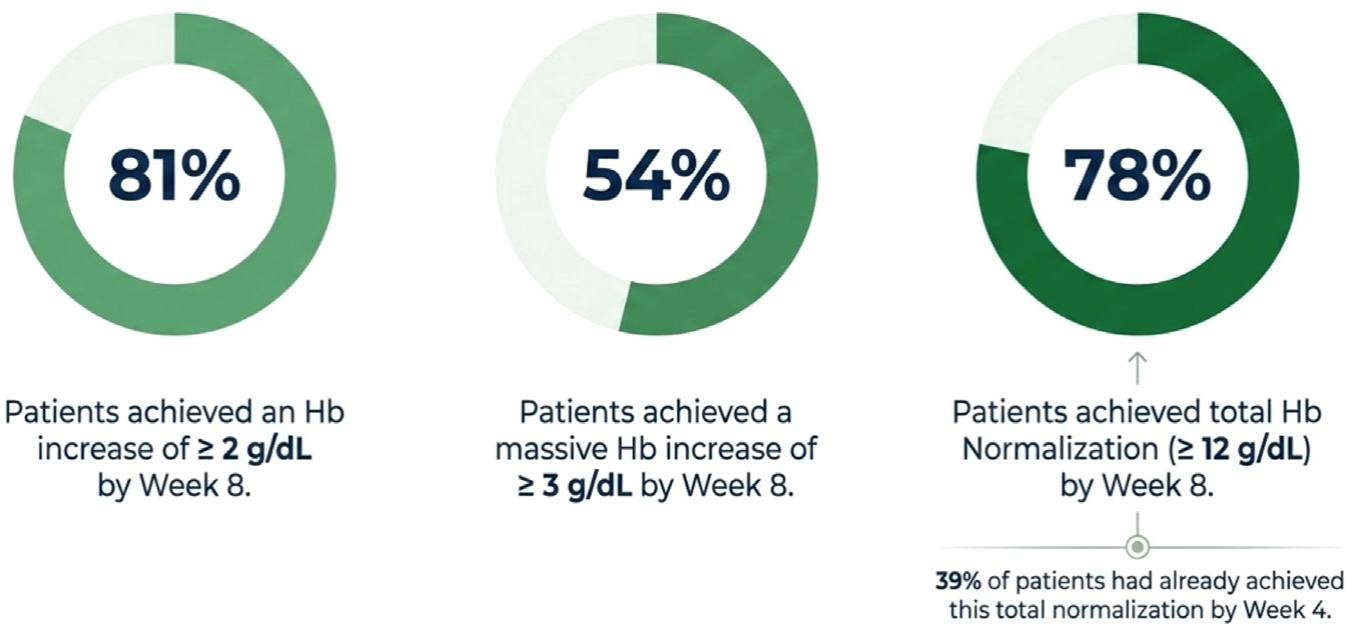
The majority of patients achieved hemoglobin gains within eight weeks. Hb: Hemoglobin.

Treatment with IFD led to significant increases in Hb and iron status markers at Weeks 4 and 8 (Table 1). The primary goal was achieved with statistical significance. Hb levels gradually increased to ≥12 g/dL over time (Fig. 1, Fig. 2). By Week 4, 69% of participants had Hb levels >2 g/dL, increasing to 81% by Week 8 (Figure 3). Similarly, 54% reached ≥3 g/dL at the latter timepoint, indicating excellent overall hematologic recovery in this cohort (Figure 4). The majority of patients achieved Hb gains within eight weeks (Figure 5). Of the 55 infusions administered, only four adverse events occurred: dizziness (n = 1), diarrhea (n = 2), and anxiety (n = 1). All were mild and self-limiting. No hypersensitivity reactions were observed at any time point; similarly, median serum phosphate levels remained stable throughout the study period (Figure 1). Mild, asymptomatic hypophosphatemia (serum phosphate <2 mg/dL) occurred in three out of 30 subjects (10%) at Week 4 (Table 2). In all three cases, serum phosphate levels returned to normal by Week 8 without intervention. There were no cases of severely reduced serum phosphate (<1 mg/dL).

**Table 1:** Baseline characteristics and laboratory evolution of the study population (n = 30).

Variable	Baseline	Week 4	Week 8	p-value
Hemoglobin (g/dL)	9.3 (8.5–10.3)	11.6 (10.8–12.5)	12.6 (11.8–13.3)	<0.01
MCV (fL)	75.9 (67.8–82.6)	82.5 (77.9–86.8)	84.2 (80.2–88.4)	<0.01
Reticulocytes (%)	1.4 (1.1–2.3)	1.4 (1.1–2.2)	1.4 (1.0–1.8)	0.90
RET-He (%)	22.9 (19.4–28.7)	30.4 (27.6–32.8)	32.9 (28.7–33.6)	<0.01
Serum Iron (µ/dL)	21 (15.5–34)	53.5 (40–88)	60 (45–85)	<0.01
Transferrin saturation (%)	6 (4–10)	20.5 (14–33)	23 (18–29)	<0.01
Serum Ferritin (µ/L)	9.5 (5.5–18)	163 (50–314)	134 (85–257)	<0.01
Serum Phosphate (mg/dL)	3.4 (2.8–3.8)	3.1 (2.6–3.7)	3.2 (2.6–3.5)	0.30

Data are presented as median with interquartile range (25th–75th percentile).

Clinical groups were compared using the Kruskal–Wallis test.

MCV: Mean Corpuscular Volume; RET-He: Reticulocyte Hemoglobin Equivalent.

**Table 2:** Serum phosphate levels in patients with transient hypophosphatemia from baseline to Week 8.

Patient	Serum phosphate level (mg/dL)		
	Basal	Week 4	Week 8
#1	2.8	1.7	2.4
#2	4.9	1.4	3.8
#3	3.4	1.4	3.7

## Discussion

Anemia is a significant public health concern, affecting roughly a quarter of the world’s population [1,2]. It primarily impacts women of reproductive age, children, and individuals with chronic conditions such as inflammatory bowel disease and kidney problems [1,3]. Although oral iron supplements are the first-line treatment, their effectiveness is often limited by gastrointestinal side effects and low adherence [5].

The FERWON-IDA trial evaluated the safety and efficacy of high-dose IFD in 1512 adult patients with IDA and intolerance or lack of response to oral iron, regardless of etiology, compared with IV iron sucrose (IS). The co-primary non-inferiority endpoint for Hb change was met. The Hb increased from baseline to Week 4 by 2.14 g/dL and to Week 8 by 2.49 g/dL, confirming non-inferiority. The authors concluded that IFD was a more convenient method of IV iron administration than IS, without sacrificing efficacy or safety [12].

Two similar randomized, open-label trials compared the safety and efficacy of IFD and IS in non–dialysis-dependent chronic kidney disease, randomizing 3050 patients to a single 1000-mg infusion of IFD or multiple infusions of IS [13,14]. The coprimary endpoints, safety, and change in Hb were assessed at Week 8 and were similar.

This Brazilian cohort represents a real-world population from a tertiary referral center. Hb levels increased significantly from baseline to Week 8, with 81% of patients achieving an increase of ≥2 g/dL and 54% achieving ≥3 g/dL. These findings are highly consistent with results from large international trials evaluating IFD [12–15], even in a smaller, more heterogeneous population (including abnormal uterine bleeding, gastrointestinal bleeding, and post-bariatric surgery).

A recent randomized clinical trial evaluated whether single-dose IV iron for the primary treatment of maternal IDA in the second trimester was superior to twice-daily oral iron. The study demonstrated that IV iron (IFD and ferric carboxymaltose) was safe and effective, reducing the incidence of low-birth-weight infants and increasing the incidence of attaining a maternal nonanemic state without the use of additional iron or blood transfusions [15]. The authors emphasize that clinical guidelines should address the potential benefit of single-dose IV iron as the primary treatment for moderate iron-deficiency anemia in pregnancy [15].

Results from the IFD trial in the United States involving over 2000 patients showed that the incidence of severe hypersensitivity reactions ranged from 0.3% to 0.8% [12,13,16]. In the present study, the treatment was widely accepted, with mild AEs occurring rarely (8%) and no moderate or severe hypersensitivity reactions [6, 7, 8,11]. These results are consistent with the contemporary understanding that modern IV iron formulations carry a low risk of severe hypersensitivity reactions.

Awareness of hypophosphatemia, a relatively common AE, is essential because of its potential consequences (e.g., bone loss, osteomalacia), especially after repeated high-dose treatment [6]. In the FERWON-IDA trial, hypophosphatemia (3.9% with IFD and 2.3% with IS) was temporary, with no cases of severe hypophosphatemia (serum phosphate <1.0 mg/dL) associated with either treatment [12]. Similarly, in the FERWONNEPHRO trial, few patients treated with IFD (3.2%) and IS (0.8%) developed hypophosphatemia, and none experienced severe hypophosphatemia [13].

In the current study, hypophosphatemia occurred in 10% of participants, was mild and asymptomatic, and self-limited (tended to resolve spontaneously within eight weeks of observation). No severe hypophosphatemia was observed. These results are consistent with published evidence showing that IFD has a significantly lower risk of clinically significant hypophosphatemia than other IV iron compounds, such as ferric carboxymaltose [12, 13, 14,16].

The results of this study demonstrate that IFD is effective for treating patients with IDA who are intolerant of or unresponsive to oral iron. While existing literature suggests benefits regarding adherence and resource utilization, the findings of this study specifically confirm the clinical effectiveness and safety of this formulation within a Brazilian real-world cohort [6, 7, 8, 9, 10]. Expanding access to modern IV iron formulations in low- and middle-income countries could help reduce ongoing disparities in anemia treatment. They may decrease the need for transfusions, hospital visits, and longer treatment durations. Such efforts are essential for developing better treatment options that benefit individuals and communities worldwide through collective actions [1, 2, 3,7,9].

Despite some limitations of this study such as a relatively small number of patients that limit statistical power and generalizability; a single-center design that may restrict diversity in patient populations and healthcare settings; a short follow-up period (eight weeks) that only captures short-term efficacy and safety rather than long-term outcomes; and the absence of a comparator arm (such as IS or ferric carboxymaltose), it still makes a meaningful contribution to the literature on IDA treatment. It provides a foundation for larger, more comprehensive research. Additionally, it adds value to the Latin American literature, which remains underrepresented in international datasets on anemia management.

## Conclusion

This study contributes to the growing evidence supporting the use of IFD as a safe and effective alternative for specific IDA populations. In this real-world clinical setting, the treatment is effective for treating patients with iron deficiency, providing rapid hematologic recovery with a favorable safety profile.

## Conflicts of interest

The authors declare institutional support (ferric derisomaltose and all laboratory tests) from Pfizer.

## Data Availability

The data that support the findings of this study are available from the corresponding author upon reasonable request.
